# Theragnostic Imaging Using Radiolabeled Antibodies and Tyrosine Kinase Inhibitors

**DOI:** 10.1155/2015/842101

**Published:** 2015-03-22

**Authors:** Mitsuyoshi Yoshimoto, Hiroaki Kurihara, Hirofumi Fujii

**Affiliations:** ^1^Division of Functional Imaging, National Cancer Center Hospital East, 6-5-1, Kashiwanoha, Kashiwa, Chiba 277-8577, Japan; ^2^Diagnostic Radiology, National Cancer Center Hospital, 5-1-1 Tsukiji, Chuo-ku, Tokyo 104-0045, Japan

## Abstract

During the past decade, the efficacy of new molecular targeted drugs such as tyrosine kinase inhibitors (TKIs) and monoclonal antibodies has been proven worldwide, and molecular targeted therapies have become the mainstream in cancer therapy. However, clinical use of these new drugs presents unexpected adverse effects or poor therapeutic effects. Therefore, we require diagnostic tools to estimate the target molecule status in cancer tissues and predict therapeutic efficacy and adverse effects. Although immunohistochemical, polymerase chain reaction (PCR) and fluorescence in situ hybridization (FISH) analyses of biopsy samples are conventional and popular for this diagnostic purpose, molecular imaging modalities such as positron emission tomography (PET) and single photon emission computed tomography (SPECT) are also useful for noninvasive estimation of gene and protein expression and drug pharmacokinetics. In this review, we introduce new radiolabeled TKIs, antibodies, and their clinical application in molecular targeted therapy and discuss the issues of these imaging probes.

## 1. Introduction

New observations regarding carcinogenesis and signal transduction pathways that regulate tumor growth, differentiation, angiogenesis, invasion, and metastasis have led to the identification of potential therapeutic targets and have accelerated molecular targeted drug development. In particular, the success of imatinib in chronic myeloid leukemia (CML) patients has strongly promoted the development of small-molecule tyrosine kinase inhibitors (TKIs). Since the United States Food and Drug Administration's approval of rituximab (Rituxan; anti-CD20 antibody) and imatinib (Gleevec; Bcr-Abl TKI), several anticancer drugs have been approved each year in the US, European Union, and Japan [1].

The antitumor mechanisms triggered by molecular targeted drugs differ from those of conventional chemotherapeutic agents. Therefore, the estimation of target molecule expression in entire tumor is required to predict therapeutic efficacy. Target molecule and target gene expressions can be evaluated using immunohistochemical, polymerase chain reaction (PCR) and fluorescence in situ hybridization (FISH) analyses of biopsy samples. However, biopsy samples contain tissues from limited regions only, whereas tumor tissue is heterogeneous. Thus, it is possible that the expression observed in biopsy samples is not representative of that in entire tumor [2, 3]. This can lead to a misunderstanding with respect to tumor characterization. Moreover, expression levels of key molecules and gene mutations require modulation during treatment. The consequent repetitive biopsies are invasive and represent a significant burden on patients.

Molecular imaging modalities such as positron emission tomography (PET) and single photon emission computed tomography (SPECT) are suitable for noninvasive estimation of gene and protein expressions and drug pharmacokinetics [4, 5]. Molecular imaging also enables detection of changes in gene and protein expressions in response to treatment in the entire tumor and could overcome the issues associated with biopsy. Therefore, PET and SPECT are the best tools in treatment strategies that combine therapeutics with diagnostics, also known as “theragnostics.”

Theragnostic imaging by using radiolabeled molecular targeted drugs provides new important insights into drug development and cancer treatment. For instance, theragnostic imaging reveals pharmacokinetics of drugs in individual patients. This allows stratification of the patients who would benefit from the drugs and identification of modified status of target molecules (expression levels and mutation status). Moreover, understanding of the pharmacokinetics is helpful to select candidate drugs in the process of drug development, resulting in reduction of development cost.

## 2. Development of Imaging Agents for Epidermal Growth Factor Receptor-Tyrosine Kinase (Figure 1)

The small molecule epidermal growth factor receptor (EGFR)-TKIs gefitinib and erlotinib have been approved for the treatment of non-small-cell lung cancer (NSCLC) and have exhibited dramatic antitumor activities. These therapeutic agents have been found to be effective primarily in patients with mutant EGFR-TK [6–8]. However, gefitinib treatment has also led to serious side effects such as interstitial lung disease [9]. In addition, the gefitinib treatment will result in acquisition of resistance usually within a year, half of whose mechanism is secondary T790M mutation of the EGFR gene [10]. These clinical findings demonstrate the need to detect mutation status of the target molecule.

**Figure 1:**
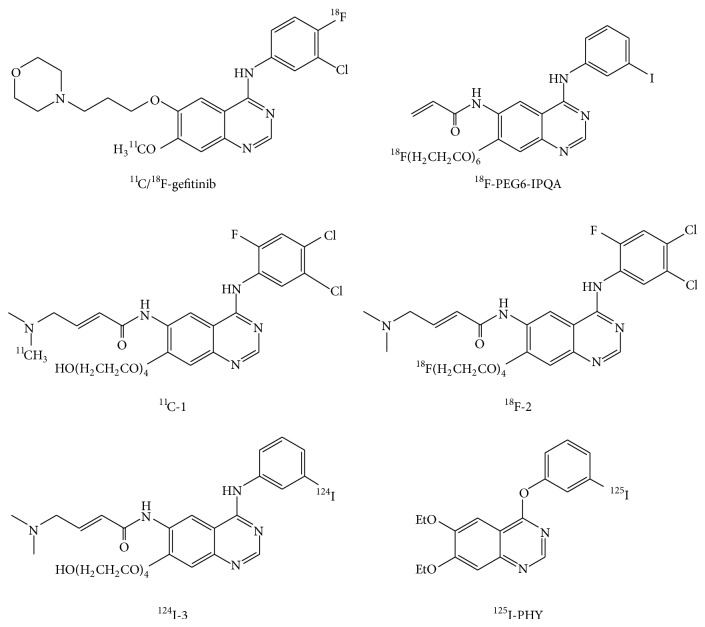
Chemical structures of the EGFR-TK imaging probes.

The simplest strategy for estimation of gefitinib sensitivity and mutation status is the use of radiolabeled gefitinib (Figure 1) [11, 12]. However, a discrepancy in specificity of radiolabeled gefitinib exists between ^18^F-gefitinib and ^11^C-gefitinib. Su et al. reported that ^18^F-gefitinib uptake* in vitro* and* in vivo* did not correlate with EGFR expression because of nonspecific binding caused by its high lipophilicity [11]. An* in vitro* uptake study indicated that high and specific ^18^F-gefitinib uptake was observed only in H3255 with mutant EGFR, but not in U87-EGFR. Unlike ^18^F-gefitinib, specific ^11^C-gefitinib uptake was observed in mice bearing murine fibrosarcoma (NFSa) [12]. However, a biodistribution study has shown that ^11^C-gefitinib uptake was low in A431 cells which exhibit high EGFR expression. Thus, radiolabeled gefitinib may not estimate EGFR expression or mutation status.

A reduction in lipophilicity might be a simple solution to overcome the nonspecific binding of an imaging probe. However, a certain level of imaging probe lipophilicity is essential for passage through the cell membrane and binding to the ATP binding pocket in the TK domain of the target molecule. A recent PET study indicated the failure of EGFR-expressing U-87MG cells to take up polyethylene glycol(PEG)-ylated anilinoquinazoline derivatives (^11^C-1, ^18^F-2, and ^124^I-3) [13]. However, PET using 4-[(3-iodophenyl)amino]- 7-(2-[2-{2-(2-[2-{2-(^18^F-fluoroethoxy)-ethoxy}-ethoxy]-ethoxy)-ethoxy}-ethoxy]-quinazoline-6-yl-acrylamide) (^18^F-PEG6-IPQA) could delineate tumors with high EGFR expression [14]. These inhibitors are irreversible (Figure 1). Although the affinities (K_D_) of these compounds for EGFR-TK are not clear, an understanding of the relationships between lipophilicity, affinity, and the binding mode (reversible or irreversible) might lead to a breakthrough in the development of TK imaging probes.

## 3. Estimation of the Mutation Status Using EGFR-TK Imaging Probes

Monitoring of EGFR-TKI sensitivity and mutation status of the target molecule is of particular interest to the field of molecular imaging. We previously reported a correlation between the tumor uptake of 4-(3-^125^I-iodo-phenoxy)-6,7-diethoxy-quinazoline (^125^I-PHY, Figure 1) and gefitinib sensitivity [15]. However, differences in tumor uptake were due partly to differences in EGFR expression and partly to nonspecific binding. Unfortunately, ^125^I-PHY could not estimate differences in mutation statuses. Yeh et al. attempted to detect a gefitinib-sensitive mutation (L858R) using ^18^F-PEG6-IPQA which selectively and irreversibly binds to mutant EGFR-TK (L858R) [14]. An* in silico* docking study revealed that the acrylamide moiety of F-PEG6-IPQA can form a covalent bond with Cys773 in the active conformation of L858R mutant kinase domain. A PET study involving ^18^F-PEG6-IPQA demonstrated high uptake in H3255 cells harboring the L858R mutation.

Memon et al. reported high and sustained ^11^C-erlotinib uptake in HCC827 cells harboring a delE746-750 mutations as compared with A549 and NCI358 cells [16]. This difference was caused by different affinities of erlotinib to EGFR-TKs [17]. A recent clinical study has supported these basic research findings. A PET study involving ^11^C-erlotinib was conducted in patients with wild-type (WT) and Exon19 deletion mutation. Although patients harboring the common L858R mutation were not included, the uptake of ^11^C-erlotinib was higher in tumors with the deletion mutation than those with WT [18]. Moreover, these ^11^C-erlotinib studies suggest that reversible inhibitors are efficient imaging probes for estimating the mutation status based on differences in binding affinity.

Unfortunately, this study has some limitation. Only a small number of patients (5 with and 5 without a mutation) were involved. Patients with other mutation (L858R and L858R/T790 M) were not included. Further investigation is needed to demonstrate that ^11^C-erlotinib PET is as efficient as the conventional mutation test using the biopsied samples.

## 4. Human Epidermal Growth Factor Receptor 2 (HER2) Imaging

Anti-HER2 therapy with trastuzumab, a monoclonal antibody, is now well established in HER2-positive breast cancer patients [19, 20]. Although HER2 expression is estimated using immunohistochemical or FISH analyses of biopsy samples, core needle biopsy is not possible for some lesions [21]. In addition, HER2 status can change during disease progression and over the course of the treatment [22, 23].

**Figure 2:**
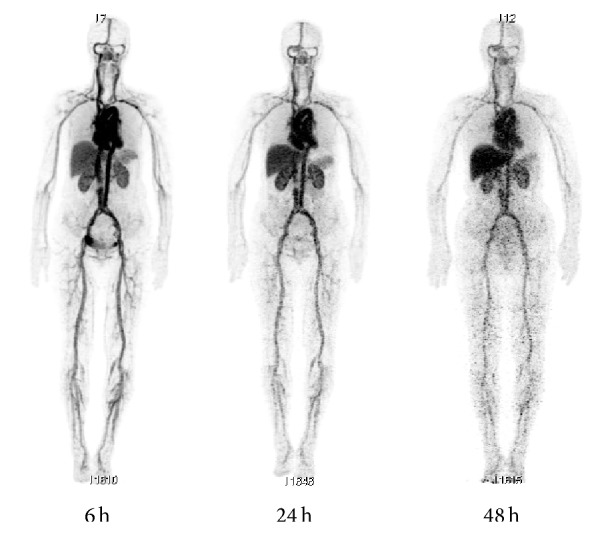
Whole-body ^64^Cu-DOTA-trastuzumab PET images at 6, 24, and 48 h after injection.

There have been many reports of the use of radiolabeled trastuzumab in animal and human studies (Figure 2) [24–28]. McLarty et al. reported that the tumor uptake of ^111^In-DTPA-trastuzumab, after correcting for nonspecific IgG accumulation and circulating radioactivity, exhibited strong nonlinear associations with the HER2 density in a mouse model of breast cancer [29]. PET with ^89^Zr-trastuzumab could detect downregulation of HER2 expression in response to afatinib treatment in a gastric cancer xenograft model [30]. Moreover, the first-in-human study of ^89^Zr-trastuzumab PET resulted in tumor visualization and quantitative tracer uptake in HER2-positive tumors [24].

A long duration of antibody circulation leads to a high background signal, and therefore a long period of time is needed to decrease the radioactivity in the blood and acquire specific images of the target tissues. Although metal radionuclides with long half-lives such as ^111^In and ^89^Zr (67.9 h and 78.4 h, resp.) are appropriate for* in vivo* imaging with radiolabeled antibodies, the use of these radionuclides results in high levels of radiation exposure. Tamura et al. reported that ^64^Cu-DOTA-trastuzumab could delineate HER2-positive lesions including brain metastases in breast carcinoma patients; further, the use of ^64^Cu (half-life = 12.7 h) could reduce the radiation exposure to 4.5 mSv versus the 18 mSv from ^89^Z-trastuzumab [24, 28]. This effective dose is similar to that of ^18^F-FDG (0.019 mSv/MBq) [31].

## 5. Vascular Endothelial Growth Factor (VEGF) Imaging

**Figure 3:**
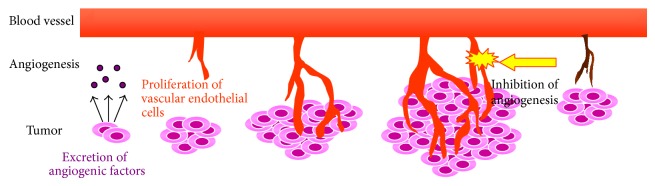
Depiction of tumor angiogenesis. Angiogenesis is an important tumor growth factor. Vascular endothelial cell proliferation is essential for the development of new blood vessels.

Antiangiogenic therapy is a cancer treatment strategy [32, 33]. Angiogenesis consists of various processes such as the secretion of angiogenic factors and the proliferation of vascular endothelial cells (ECs; Figure 3). Bevacizumab is a monoclonal anti-VEGF antibody that inhibits VEGF-stimulated EC proliferation [34, 35]. Vandetanib, a VEGFR-TK inhibitor, directly inhibits EC proliferation because VEGF expression is upregulated within tumor tissues [36, 37]. Antiangiogenic drugs directly or indirectly inhibit EC proliferation followed by tumor suppression. Therefore, to decide the therapeutic plan using antiangiogenic drugs, we should estimate the expression level of the target molecule, but not the biological activity of tumor cells.

Molecular imaging could be a powerful tool for estimating the VEGF content within tumor tissues. Enzyme-linked immunosorbent assays of tissue samples measure not only extracellular but also intracellular VEGF content. The first clinical study of ^89^Zr-bevacizumab PET conducted in breast cancer patients indicated a correlation between the maximum standard uptake values of the tracer and VEGF-A expression in the tumors [38]. Chang et al. reported that ^64^Cu-NOTA-bevacizumab could be used to evaluate a decrease in the expression of VEGF caused by everolimus, a mammalian target of rapamycin inhibitor [39].

## 6. Other Kinase Imaging with Radiolabeled Small Molecules

New kinase inhibitors have been labeled with ^11^C or ^18^F; these include ^11^C-vandetanib, ^18^F-SKI696 (an analogue of imatinib), and ^18^F-sunitinib [40–46]. However, their chemical structures must be modified for compatibility with the imaging probes. The biodistribution of ^11^C-sorafenib indicated high radioactivity in the liver and slow blood clearance [46]. The tumor uptake of ^11^C-sorafenib was lower than its radioactivity level in blood. In view of selectivity, we could not deduce which kinases contribute to tumor uptake because sorafenib is a multikinase inhibitor. The properties of the oral drugs that are high bioavailability and high blood concentration may be unfavorable for imaging probes. Therefore, optimization of chemical structures would be required for use of kinase inhibitors for imaging probes. The use of radiolabeled kinase inhibitors may be limited to the purpose of estimating pharmacokinetics and drug concentrations.

## 7. Conclusion

Targeted therapy is becoming the mainstream in the field of cancer therapy, and the development of new targeted drugs is increasing. However, the therapeutic effects of the agents remain limited, and patients who benefit from targeted therapy represent a fraction of all patients. Molecular imaging plays two roles in targeted therapy. The first role is the estimation of features of the target molecules such as the expression level and mutation status, thus allowing patient stratification. The second role is the elucidation of pharmacokinetics and measurement of drug concentrations in the tissues. Drug radiolabeling is sufficient for the latter purpose, whereas the former requires imaging probes for the acquisition of informative images. We should readily try to optimize the chemical structures of kinase inhibitors for use as imaging probes instead of antibodies. We believe that advances in imaging probes will contribute to our understanding of pharmacokinetics/pharmacodynamics, drug development, and therapy planning.
